# Comparison of Rectal and Intraoperative Findings in Horses with Colic

**DOI:** 10.3390/ani16142175

**Published:** 2026-07-13

**Authors:** Marie-Therese Schlote, Roswitha Merle, Sophie McCullagh, Bartlomiej Obrochta, Simon Hennessy, Miranda Dosi, Heidrun Gehlen

**Affiliations:** 1 Institute of Equine Internal Medicine, Freie Universität Berlin, Oertzenweg 19B, 14163 Berlin, Germany; 2 Institute of Veterinary Epidemiology and Biostatistics, School of Veterinary Medicine, Freie Universität Berlin, 14163 Berlin, Germany; roswitha.merle@fu-berlin.de; 3 Royal Dick School of Veterinary Studies, University of Edinburgh, Scotland EH25 9RG, UK; 4 Equine Hospital, Husaby Hästklinik, 533 95 Götene, Sweden; 5 Anglesey Lodge Equine Hospital, R51HK85 Kildare, Ireland

**Keywords:** laparotomy, abdominal pain, colon torsion, rectal examination, equine colic

## Abstract

Per rectum examination (PRE) is commonly used in the triage of horses with acute abdominal pain, despite reports of associated risk factors for both horses and personnel, but its diagnostic accuracy is questionable. This multicenter, observational study aimed to evaluate the agreement between PRE findings and surgical findings obtained via exploratory laparotomy in horses with colic. A total of 201 cases from four equine referral hospitals across Europe were included. Rectal findings were categorized as primary (most likely cause of colic) or additional. Agreement with surgical findings was assessed using kappa statistics. The influence of examiner demographic data (age, gender, and qualification status) on diagnostic accuracy was also evaluated. The agreement between primary rectal and primary surgical diagnosis was 57.2% (115/201; kappa coefficient, 0.490; 95% confidence interval, 0.394–0.586). When both primary and additional findings were considered, agreement increased to 71.6% (144/201, kappa coefficient, 0.720; 95% confidence interval, 0.156–0.927; 65.1–77.4%). The highest agreement was seen in cases of gas-distended large colon (90.3%), small intestinal distension (75.8%), and left dorsal displacement of the large colon (66.7%). Detection of small intestine distension in PRE had the highest sensitivity (85.7%) and negative predictive value (92.8%) among the evaluated parameters. Examiner demographics had no significant impact on diagnostic accuracy. PRE is a valuable but imperfect diagnostic tool. Given the dynamic nature of colic and possible changes between examination and surgery, some discrepancies are expected. Other variables, such as horse size or the examiner’s hand dominance, were not investigated in this study but may have had an impact on the observed outcomes. Overall, PRE remains a practical, accessible technique that provides clinically useful information regardless of examiner experience.

## Simple Summary

Per rectum examination (PRE) has been anecdotally associated with severe complications in equine patients and poses potential risks to the veterinarian. Despite this, it remains a standard component of the clinical assessment for horses presenting with acute abdominal pain even though its diagnostic accuracy is known to be questionable. The objective of this study was to evaluate the agreement between findings obtained via PRE and definitive diagnoses established during exploratory laparotomy in horses with colic. We hypothesized that diagnostic accuracy would be positively associated with examiner experience, and that a high level of agreement would exist between rectal examination findings and surgical diagnoses.

## 1. Introduction

Colic is the most common emergency condition encountered in equine veterinary practice, with reported incidence rates ranging from 3.5 to 26 cases per 100 horses at risk per year [1,2,3,4,5]. A thorough diagnostic evaluation is essential in horses presenting with signs of abdominal pain, with per rectum examination (PRE) being one of the most fundamental and routinely performed diagnostic procedures [6]. PRE allows palpation of structures in the caudal abdominal cavity, including portions of the large colon, small colon, cecum, caudal margin of the spleen, caudal pole of the left kidney, aorta, mesenteric root, bladder and peritoneal surfaces. Additionally, it facilitates assessment of the reproductive tract in mares and the inguinal rings in stallions [7,8]. Under pathological conditions, it may also be possible to palpate the stomach and segments of the small intestine [9,10]. Despite its widespread use, the diagnostic accuracy of PRE has not been systematically compared to intraoperative findings, and the sensitivity and specificity of PRE in identifying surgically confirmed lesions remains unquantified. Understanding the likelihood that a PRE yields a correct diagnosis would significantly support clinical decision-making.

Furthermore, the effects of examiner-related factors that may influence diagnostic agreement—such as age, experience level, and professional qualification—are poorly understood. Clarifying these aspects is critical to improving clinical decision-making and refining training protocols for veterinarians managing equine colic.

PRE is not without risk and reported complications include rectal tears, mucosal irritation, and injury to the examiner [7,8,11,12]. In a survey of veterinary practitioners, PRE was the second-most necessary diagnostic test for the primary evaluation of colic in horses, with response to analgesia deemed the most important. The three main reasons why veterinarians did not perform PRE were safety concerns for the examiner, uncooperative behaviour of the horse and the fact that PRE would not influence treatment decisions [13]. Despite its limitations, PRE remains particularly useful in identifying horses that may require surgical intervention and is recommended in all colic cases, provided safety is maintained [14]. It is important to note that abnormalities detected during preoperative rectal examination do not necessarily indicate the need for surgical intervention. Findings such as unexplained intestinal distension or abnormal positioning, particularly when no definitive diagnosis can be established or when accompanied by intractable pain, may increase suspicion of a surgical lesion. Conversely, conditions such as large colon impactions or tympany are frequently amenable to medical management. Whilst PRE is a valuable diagnostic tool, its sensitivity for determining the necessity of surgery remains limited [15]. Rectal findings that are more likely to point to a condition requiring surgery include: inguinal herniation, tightly coiled loops of small intestine, a distended and edematous colon, a tightly filled cecum with fluid ingesta, and severe distention of any intestinal segment [15,16]. Accurate interpretation of PRE findings is essential, as they often guide further diagnostic and therapeutic decisions [7]. This study aimed to evaluate the agreement between rectal findings and surgical findings in horses undergoing exploratory laparotomy for colic. We evaluated the diagnostic performance—including sensitivity, specificity, positive and negative predictive values—of the most frequently identified conditions on rectal examination.

We hypothesized that the accuracy of rectal diagnosis would improve with the examiner’s age and qualification level, with board-certified specialists expected to demonstrate greater diagnostic concordance compared to newly graduated veterinarians, residents, or those without advanced qualifications. In addition, we hypothesized that small intestinal distention and right dorsal displacement of the large colon would have the highest diagnostic accuracy via rectal examination.

## 2. Materials and Methods

### 2.1. Data Collection

Inclusion criteria were: horses over one year of age, presentation to a participating referral hospital for signs of abdominal discomfort (colic), and performance of a PRE prior to exploratory laparotomy. Horses presenting with abdominal pain who did not undergo PRE before surgery were excluded from the study. Data collection was conducted prospectively across four equine referral hospitals in Europe (Germany, UK, Finland and Ireland) from January 2023 to November 2024. All horses had at least one PRE at the referral hospital prior to laparotomy. PRE results from the referring veterinarians were not included. The last rectal examination prior to laparotomy was used in the analysis. The surgeons who performed the laparotomies were not involved in performing PREs.

### 2.2. Examiner

Demographic data of the examiner, including age, gender, and qualification status, as well as rectal examination findings, were recorded anonymously immediately following the PRE and prior to the horse undergoing laparotomy. Age was categorized into six groups: Group 1 (20–30 years), Group 2 (31–40 years), Group 3 (41–50 years), Group 4 (51–60 years), Group 5 (61–70 years), and Group 6 (>70 years). Gender was classified into three categories: female, male, and other. Qualification status of the examiner was grouped into six categories: recently graduated (≤2 years post-graduation), resident, diplomate, Fachtierarzt (German board certification in advanced veterinary practice), certificate in advanced veterinary practice approved, or no additional qualification. For the purposes of this study, Fachtierarzt was considered equivalent to a UK certificate. No further differentiation was made between specialties (e.g., ECEIM, ECVS, ECVDI). When an examiner held multiple qualifications, only the highest level was recorded, with diplomate status considered the highest.

### 2.3. Rectal Findings

**Figure A1:**
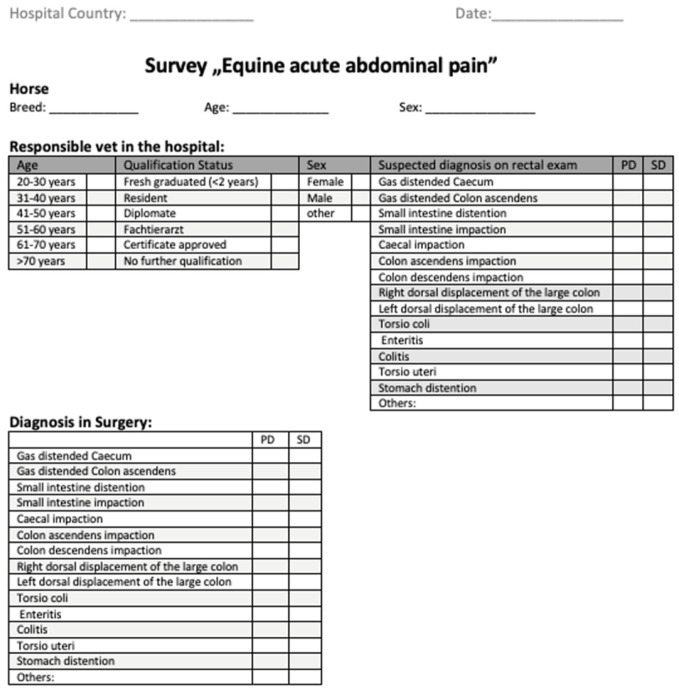
Acute abdominal pain survey.

Rectal diagnoses were categorized into 15 predefined groups: gas-distended cecum, gas-distended large colon, small intestinal distention, small intestinal impaction, cecal impaction, large colon impaction, small colon impaction, right dorsal displacement of the large colon (RDDLC), left dorsal displacement of the large colon (LDDLC), large colon torsion, enteritis, colitis, uterine torsion, distended stomach, and “others.” For entries categorized as “others,” a written description of the finding was required. In cases where both the rectal and surgical diagnoses fell into the “others” category, the handwritten notes were compared. If the descriptions did not match, the case was classified as a disagreement. For each case, the examiner identified one suspected primary finding—the most likely cause of abdominal discomfort—and could list additional findings if applicable. Additional findings were defined as any clinically relevant abnormalities detected during the per rectum examination that were not part of the primary finding but were documented by the examiner. Only one primary diagnosis was allowed per horse, though multiple additional findings could be recorded. The data collection form is included in Appendix A, Figure A1.

### 2.4. Surgical Findings

All horses included in the study underwent exploratory laparotomy. Surgical diagnoses were categorized into the same 15 predefined groups as those used for rectal examination findings. For each case, the attending surgeon identified a single primary diagnosis—defined as the most likely cause of the observed abdominal discomfort. Additional findings could be recorded if present; however, only one primary diagnosis was assigned per horse. The indications for laparotomy varied and were not based solely on physical PRE findings. Surgical intervention was performed in cases where horses demonstrated persistent signs of abdominal pain despite analgesic treatment, exhibited abnormal peritoneal fluid characteristics on abdominocentesis, or showed cardiovascular compromise, among other criteria.

### 2.5. Statistical Analysis

Data were analyzed using IBM SPSS Statistics for Windows Version 29 (Armonk, NY, USA: IBM Corp). Continuous data were summarized as mean ± SD and median; categorical data were summarized as counts and percentages including 95% confidence intervals (95% CIs). Cohen’s kappa was used to express agreement between PRE and the surgical diagnosis during laparotomy, also including 95% CIs. Two approaches were used to explore agreement: first, whether there was agreement in the primary diagnosis (most likely the cause of colic) both of PRE and surgery; second, whether there was agreement in any finding that was recorded in both PRE and surgery. The influence of age, sex, and qualification status on the agreement of rectal diagnosis with surgical diagnosis (both approaches) were evaluated using multivariable logistic regression models with agreement (yes/no) as the dependent variable. Values of *p* < 0.05 were considered significant. Sensitivity (true positives/[true positives + false negatives]), specificity (true negatives/[true negatives + false positives]), positive predictive value (true positives/[true positives + false positives]) and negative predictive value (true negatives/[true negatives + false negatives]) were calculated. Agreement was interpreted according to Cohen’s kappa coefficient as follows: <0 indicating no agreement, 0.00–0.20 slight agreement, 0.21–0.40 fair agreement, 0.41–0.60 moderate agreement, 0.61–0.80 substantial agreement, and 0.81–1.00 almost perfect agreement.

## 3. Results

### 3.1. Horses

A total of 202 horses were enrolled in this multicenter observational study. One horse was excluded from the final analysis due to incomplete data, resulting in 201 horses being evaluated. All analyzed horses were presented to referral hospitals with clinical signs of abdominal discomfort and underwent at least one per rectum examination at the referral hospital prior to exploratory laparotomy. The study population comprised a mixed cohort of mares (*n* = 98), geldings (*n* = 93) and stallions (*n* = 10), representing 23 different breeds: Warmbloods, Thoroughbreds, Icelandic horses, Finnhorses, ponies, Haflingers, Arabians, Connemaras, Friesians, Fjord horses, Irish Draughts, Warmblood-crosses, American Quarter Horses, American Paint Horses, Konik horses, Tarpans, Belgian Drafts, Lusitanos, Clydesdales, Welsh Cobs, Irish Tinkers, and Irish Tinker-crosses. The horses ranged in age from 1 to 30 years, with a median age of 10.3 years.

### 3.2. Examiner

Per rectum examinations were performed by 153 female and 48 male veterinarians. The veterinarians performing the PREs were likely involved in the assessment of more than one horse included in the study. Veterinarians’ age distribution was as follows: 72/201 were aged 20–30 years, 93/201 were 31–40 years, 32/201 were 41–50 years, and 4/201 were 51–60 years. The proportion of recently graduated examiners was (≤2 years post-graduation) 9/201, 67/201 veterinarians were residents, 38/201 were diplomates, 28/201 held a certificate in advanced veterinary practice or Fachtierarzt, and 59/201 had no further post-graduate qualifications. Statistical analysis revealed no significant effect of examiner age (*p* = 0.511), qualification (*p* = 0.094), or gender (*p* = 0.636) on overall agreement between any rectal and surgical diagnoses (global *p* = 0.160).

### 3.3. Rectal Findings

The agreement between primary rectal and primary surgical diagnosis was 57.2% (115/201; 95% CI: 50.3–63.9%). Cohen’s kappa coefficient was 0.490 (95% CI: 0.394–0.586, *p* < 0.001), indicating moderate agreement.

The prevalence of rectal and surgical findings is listed in Table 1. The agreement between any rectal diagnosis and the primary surgical diagnosis was 58.7% (118/201, 95% CI: 51.8–65.3%). PRE findings of a gas-distended cecum (100%; 2/2), a gas-distended large colon (75%; 3/4), small intestinal distention (83.3%; 35/42), and left dorsal displacement of the large colon (66.7%; 12/18) showed the highest agreement. The agreement between any rectal and any surgical diagnosis was 71.6% (144/201, 95% CI 65.1–77.4%). Cohen’s kappa coefficient was 0.720 (95% CI: 0.156–0.927, *p* < 0.001), indicating substantial agreement. The agreement of rectal and surgical diagnoses compared with each other is summarized in Table 2.

Out of 201 horses, a total of 67 horses had at least one additional finding on PRE. The three most common additional findings on PRE were a gas-distended cecum (10/67), a gas-distended large colon (28/67), and large colon impaction (18/67). Small intestine distention (5/67), small colon impaction (1/67), right dorsal displacement of the large colon (3/67), large colon torsion (1/68), and one case categorized as “others” were noted less frequently. Primary or additional rectal findings in the category “other” were large colon wall edema, scrotal hernia, abscess, strangulating small colon, and no abnormal findings on PRE. Additional results are presented in Table 2. As the assignment of primary PRE and surgical diagnoses was subject to examiner interpretation, the overall agreement between any PRE and surgical findings (last column of Table 2) should be considered the most informative measure.

During surgery, 129 of the 201 horses had at least one additional diagnosis. The three most common additional findings during surgery were large colon impaction (41/129), a gas-distended large colon (27/129), and a distended small intestine (20/129). Primary or additional surgical diagnoses categorized as “others” were equine grass sickness, scrotal hernia, strangulating small colon, mesenteric torsion, adhesions of the small intestine or large colon, large colon wall edema, typhlitis, impaction of the ampulla coli, hypoplasia of the spleen, abscess, and no abnormal findings during surgery. For the four most common findings during explorative laparotomy, we calculated sensitivity, specificity, and positive and negative predictive values. The results are presented in Table 3.

## 4. Discussion

Per rectum examination remains the gold standard for the initial assessment of horses presenting with abdominal discomfort and should be performed in all colic cases unless it poses a safety risk to either the horse or the veterinarian [1,16].

Large colon impactions were generally well detected on PRE. Large colon impactions located in the caudal abdomen are typically more accessible and therefore easier to identify on PRE [17]. In contrast, large colon torsions were frequently underdiagnosed and were often misinterpreted as right dorsal displacement of the large colon (RDDLC). This is a clinically relevant distinction, as a large colon torsion is a surgical emergency, whereas RDDLC may respond to medical treatment in the absence of complications such as unrelenting pain, elevated lactate, or ultrasonographic evidence of colon wall thickening. These findings in a case diagnosed with an RDDLC could be suggestive of an unidentified large colon torsion. In cases of 360° torsion, the colon may lie in an anatomically deceptive position, despite complete mesenteric vascular occlusion [18]. If the torsion is incomplete, a tight taenia may be palpable, which can be mistaken for an RDDLC [19]. Careful assessment of taenia direction may help differentiate between these two conditions [20]. PRE is limited to the caudal abdomen and may be compromised by excessive gas accumulation [21]. Sensitivity for RDDLC was 62.7%, which was higher than the sensitivity for large colon torsions (44.8%) but the negative predictive value was higher for large colon torsions (80.7% vs. 73.5%). Accurate preoperative identification of large colon pathology can be challenging. In such cases, ultrasonography has been shown to correlate well with both small and large colon lesions and can serve as a valuable adjunctive diagnostic modality [22]. In contrast, small intestinal distension demonstrated good agreement between rectal examination and surgical findings and frequently represented a clear diagnosis. The relatively high sensitivity (85.7%) and negative predictive value (92.8%) indicate that PRE may be useful for reliably ruling out small intestinal distension. The timing of PRE and localisation of the small intestinal obstruction might influence these results significantly. Turner at al. [23] reported small intestine distention in only 8/15 horses with epiploic foramen entrapment, suggesting that this specific cause of small intestinal strangulation distension might take longer to develop and therefore remain initially undetected.

Although it was hypothesized that examiner age, sex, or level of qualification might influence diagnostic accuracy, no significant differences were identified. This observation should be interpreted with caution, as it may, in part, reflect the relatively small number of diplomates and certificate holders included in the study. In addition, the specific specialty of residents and diplomates (e.g., internal medicine vs. surgery) was not recorded, which further limits interpretation regarding the potential influence of specialized training on diagnostic performance. As individual veterinarians may have examined more than one horse, some degree of variability in examiner experience cannot be excluded and may have influenced the overall performance measures, particularly if certain individuals contributed a higher number of cases.

We acknowledge that age is not a perfect proxy for clinical experience. Although older examiners might be presumed to have more experience, age alone does not equate to time in practice. Collecting data on years of clinical experience would have provided a more accurate measure but was omitted to minimize survey complexity and missing data.

There was no significant difference in the agreement between men and women. While some studies suggest that men may have an advantage in three-dimensional visualization, our findings did not reveal any sex-based differences [24,25,26]. This result may be influenced by the relatively small number of male examiners included in the study. This study did not account for examiner handedness, which may influence the ease of palpating certain intra-abdominal structures [27,28]. For example, veterinarians who palpate with their left hand may access right-sided abdominal organs, such as the cecum, more easily, while veterinarians who palpate with their right hand may find the left side more accessible. Examiner body size relative to the horse may also influence diagnostic accuracy. A smaller veterinarian examining a large horse may be limited in reach, potentially missing cranially located lesions such as small intestinal distention or impactions. Although steps or platforms can be used to mitigate height differences, arm length may still limit examination depth. Conversely, veterinarians with larger arms may also face limitations in performing a thorough palpation on smaller horses, due to patient safety considerations. Our findings support the value of PRE as a practical, though imperfect, diagnostic tool. Colic is a dynamic condition, and changes occurring between the last PRE and surgical intervention (e.g., attempted medical management or movement) may partly explain diagnostic discrepancies. Due to the dynamic nature of the disease process, the diagnosis made by the referring veterinarian may differ from that made by the veterinarian at the referral hospital. Rectal findings may also change over time. This would be of interest for future studies but was out of scope for the current study.

Finally, it should be noted that only horses undergoing laparotomy were included, which may introduce selection bias. Cases successfully managed medically—particularly impactions—are likely underrepresented in the present study. Given that these conditions are typically more easily detected during preoperative PRE, a greater number of such cases might have contributed to higher agreement rates between PRE and surgical findings [8].

## 5. Conclusions

In this study, right dorsal displacement of the large colon and small intestinal distention demonstrated the highest sensitivity of diagnostic agreement between PRE and surgical findings. Small intestinal distension and large colon torsion showed the highest negative predictive values with 92.8% and 80.7% of cases, respectively. Statistical analysis revealed no significant effect of examiner age, qualification, or gender on overall agreement between any rectal and surgical diagnoses.

Despite this, clinical decision-making should not rely solely on PRE. Additional diagnostic modalities, including abdominal ultrasonography, abdominocentesis, and hematologic evaluation, should be incorporated to guide comprehensive and evidence-based treatment planning.

## Figures and Tables

**Table 1:** Frequency of primary and additional diagnoses obtained from PRE and intraoperative findings in equine colic cases. “Primary” refers to the main diagnosis responsible for clinical signs, while “Additional” includes concurrent findings.

Diagnosis	Prevalence in Primary PRE Diagnosis	Prevalence in Additional PRE Diagnosis	Total Prevalence in PRE Diagnosis	Prevalence in Primary Surgical Diagnosis	Prevalence in Additional Surgical Diagnosis	Total Prevalence in Surgical Diagnosis
Gas-distended cecum	5	10	15	2	16	18
Gas-distended large colon	21	28	49	4	27	31
Small intestine distention	54	5	59	42	20	62
Small intestine impaction	2		2	8		8
Cecal impaction	1		1	1	1	2
Large colon impaction	12	18	30	9	41	50
Small colon impaction	2	1	3	3	2	5
Right dorsal displacement of large colon	55	3	58	59	10	69
Left dorsal displacement of large colon	19		19	18		18
Large colon torsion	14	1	15	29	1	30
Enteritis	1		1	5	1	6
Stomach distention					3	3
Others	15	1	16	21	7	28
Total	201	67	268	201	129	330

**Table 2:** Agreement between preoperative PRE and surgical (SX) diagnoses in horses with colic, expressed as proportions of matching primary and additional findings. (Primary SX = primary diagnosis identified during surgery; Any SX = primary and additional surgical findings; Primary PRE = Primary diagnosis based on preoperative rectal examination; Any PRE = primary and additional findings detected during PRE).

Diagnosis	Primary PRE vs. Primary SX *n*/N (%)	Primary SX vs. Any PRE *n*/N (%)	Primary PRE vs. Any SX *n*/N (%)	Any SX vs. Any PRE *n*/N (%)
Gas-distended cecum	2/5 (40.0)	2/2 (100)	2/5 (40.0)	7/18 (38.9)
Gas-distended large colon	2/21 (9.5)	3/4 (75.0)	8/21 (38.1)	28/31 (90.3)
Small intestine distention	35/54 (64.8)	35/42 (83.3)	43/54 (79.6)	47/62 (75.8)
Small intestine impaction	82/2 (100)	2/8 (25.0)	2/2 (100)	2/8 (25.0)
Cecal impaction	0/1 (0)	0/1 (0)	0/1 (0)	0/2 (0)
Large colon impaction	3/12 (25.0)	3/9 (33.3)	9/12 (75.0)	24/50 (48.0)
Small colon impaction	1/2 (50.0)	1/3 (33.3)	2/2 (100)	2/5 (40.0)
Right dorsal displacement of large colon	37/55 (67.3)	37/59 (62.7)	39/55 (70.9)	39/69 (56.5)
Left dorsal displacement of large colon	12/19 (63.2)	12/18 (66.7)	12/19 (63.2)	12/18 (66.7)
Large colon torsion	13/14 (92.9)	13/29 (44.9)	13/14 (92.9)	13/30 (43.3)
Enteritis	1/1 (100)	1/5 (20.0)	1/1 (100)	1/6 (16.7)
Stomach distention				0/3 (0)
Others	7/15 (46.7)	7/21 (33.3)	7/15 (46.7)	9/18 (50.0)

**Table 3:** Accuracy of the diagnoses obtained by preoperative PRE in comparison with lesions identified at exploratory laparotomy in horses with colic, including prevalence, sensitivity, and positive and negative predictive values for the four most common conditions.

Diagnosis	Prevalence (%)	Sensitivity (%)	Specificity (%)	Positive Predictive Value (%)	Negative Predictive Value (%)
Small intestinal distention	20.9	85.7	48.4	30.5	92.8
Right dorsal displacement	29.3	62.7	43.0	31.4	73.5
Left dorsal displacement	9.0	66.7	42.1	10.2	92.8
Large Colon torsion	14.4	44.8	39.0	11.0	80.7

## Data Availability

Data is unavailable due to privacy but can be provided on request.

## Abbreviations

The following abbreviations are used in this manuscript:

CI	Confidence interval
PRE	Per rectum examination
ECEIM	European College of Equine Internal Medicine
ECVS	European College of Veterinary Surgery
ECVDI	European College of Veterinary Diagnostic Imaging
PD	Primary diagnosis
SD	Secondary diagnosis
SX	Surgery
RDDLC	Right dorsal displacement of the large colon
